# Biphasic synovial sarcomas of the liver: a case report and literature review

**DOI:** 10.1186/s13000-022-01233-4

**Published:** 2022-06-02

**Authors:** Defeng Liang, Lingyu Meng, Shanshan Wang, Dan Yi, Yahui Liu

**Affiliations:** Department of Hepatobiliary and Pancreatic Surgery, The First Hospital of Jilin University, Changchun, 130000 China

**Keywords:** Synovial sarcoma, Liver, Diagnosis, Case report

## Abstract

**Background:**

Synovial sarcoma is a soft tissue sarcoma of temporarily unknown histologic origin with the ability for biphasic differentiation, occurring mostly in the vicinity of large joints of the extremities. Synovial sarcoma that originates in the liver is extremely rare. Only 7 cases have been reported in the domestic and international literature.

**Case presentation:**

We report an 11-year-old female patient who underwent partial hepatectomy for a liver mass. Microscopically, she was diagnosed with hepatic biphasic synovial sarcoma. Cytogenetic examination revealed the fusion gene SS18-SSX1 (+), which confirmed the diagnosis.

**Conclusion:**

Synovial sarcoma of the liver is a rare malignancy that is difficult to diagnose. Confirmation of diagnosis is based on histopathological assessment combined with immunohistochemical staining and, if necessary, cytogenetic aids.

**Supplementary Information:**

The online version contains supplementary material available at 10.1186/s13000-022-01233-4.

## Introduction

Synovial sarcoma (SS) is a rare but highly malignant soft tissue sarcoma, accounting for approximately 5–10% of soft tissue sarcomas [1]. However, it does not originate in the synovium, and most current studies suggest that it may originate from primitive mesenchymal cells in mesenchymal tissue [2]. The most important feature of synovial sarcoma is the ability to biphasically differentiate into the epithelium and mesenchyme, which can be divided into three subtypes: biphasic, unidirectional and hypodifferentiated [1]. It most frequently occurs in the large joints and surrounding soft tissues of the extremities. The incidence of synovial sarcoma peaks in young adults, and the median age of onset is approximately 35 years [2–4]. The etiology and pathogenesis of synovial sarcoma are not clear. However, studies have found that more than 90% of synovial sarcomas have chromosomal translocation t(X;18) (p11.2; q11.2) and produce the fusion gene SYT-SSX [5], which can be an important diagnostic basis for this disease and can be detected by fluorescence in situ hybridization (FISH) and reverse transcription polymerase chain reaction (RT–PCR). However, whether this chromosomal translocation is the cause of tumorigenesis needs further confirmation.

## Case

**Fig. 1:**
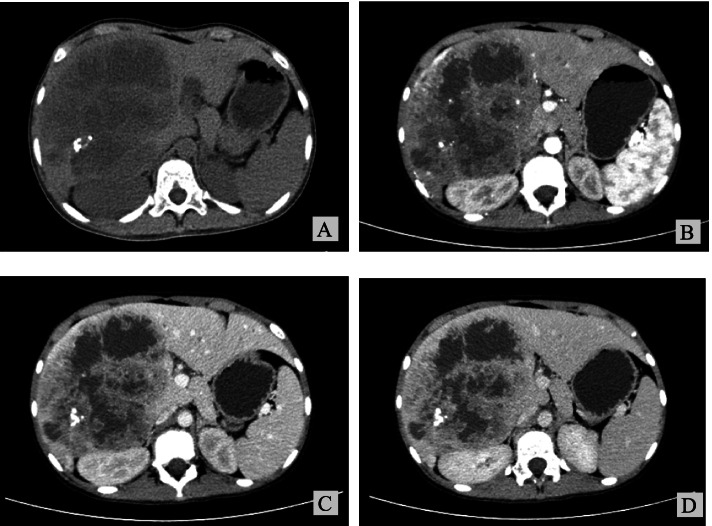
Preoperative CT. **1A** Plain CT scan shows a large mass in the right lobe of the liver. **1B** No internal enhancement was observed in the arteriolar mass on enhanced CT, with multiple cystic changes, faint drifting signs, and obvious enhancement of the surrounding liver parenchyma. **1C** Portal vein phase on enhanced CT. **1D** Delay period of enhanced CT

**Fig. 2:**
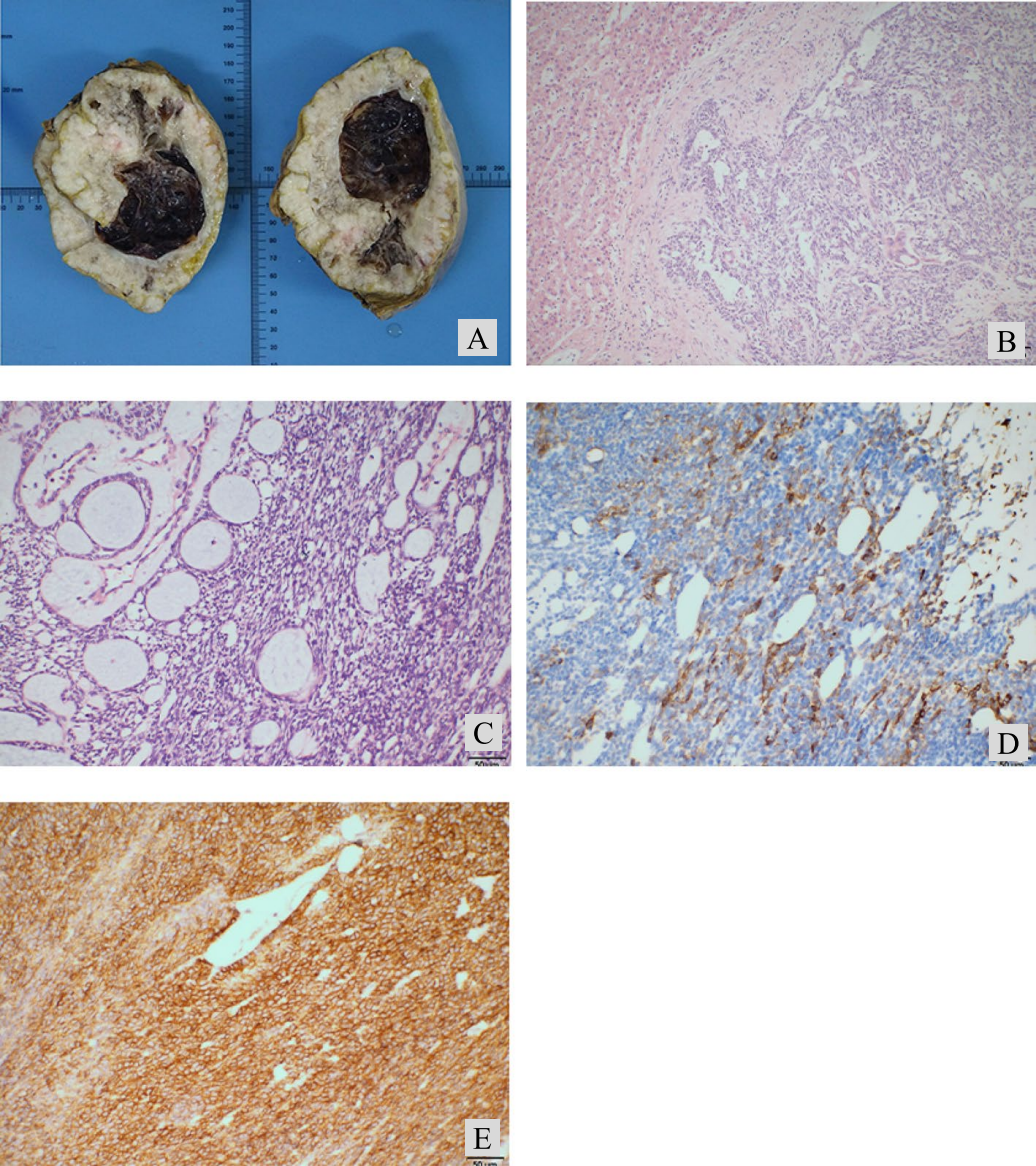
Postoperative pathological and immunohistochemical staining results. **2A** Mass specimen. **2B** Microscopically, the mass was composed of spindle cells and epithelioid cells . HE 100×. **2C** Some areas in which hemangiopericytoma blood vessels form can be observed. HE 200×. **2D** Tumor cells were positive for CK-pan. IHC 200×. **2E** Tumor cells were positive for CD99. IHC 200×

An 11-year-old female patient was admitted to the hospital on July 29, 2021 with the chief complaint of “intermittent nausea, vomiting and abdominal pain for 6 years, aggravated for 2 months”. The patient was born at full-term, had an uneventful delivery and was previously healthy. Physical examination indicated that the liver area was slightly inflated, and a liver mass could be palpated 3 cm below the rib cage, with a hard texture and positive tenderness to touch. Laboratory tests indicated that the white blood cell count was 12.92 × 10^9/L, ESR was 44 mm/1H, and tumor markers and the stool routine examination were normal. An abdominal angio CT suggested a giant low-density mass in the right lobe of the liver of approximately 13.6 × 11.2 cm in size (Fig. 1A), with a CT value of approximately 5–30 HU, and multiple cystic changes within it, with honeycomb, lava cavity-like changes and faintly visible fluttering signs with calcification. In the arterial phase, no enhancement was seen within the mass, the edges were unevenly enhanced, and the surrounding liver parenchyma was significantly enhanced (Fig. 1B). In the venous and equilibrium phases (Fig. 1C and D), it was poorly visualized. The imaging diagnosis considers hepatic echinococcosis but cannot exclude the possibility of malignancy. Surgery was planned. Before surgery, the volume of the mass measured by CT 3D reconstruction was approximately 1421 cm^2^, the total volume of the patient’s liver was approximately 2063 cm^2^, and the volume of the left liver was approximately 415 cm^2^. If the right half of the liver was removed, the functional liver volume removed was approximately 37%. In addition, the liver function evaluation results showed that the retention rate of indocyanine green was 2.5% in 15 minutes. These results indicated that the patient had good liver function and was expected to tolerate the surgery. Therefore we operated on 11 August 2021. During the surgery, we observed a large mass in the right lobe of the liver that was confined to the liver and did not break through the peritoneum. Exploration of the other abdominal organs showed no abnormalities, so we removed the intact mass together with the right half of the liver. The postoperative pathology specimen (Fig. 2A) showed a large grayish-white stromal area within the resected liver tissue, measuring approximately 18 cm × 13 cm × 9 cm, with a localized hardness around the stromal area and a central cystic cavity, 7 cm in diameter, containing a jelly like substance. Microscopically, the swelling was consisted of both spindle cells and epithelioid cells (Fig. 2B). The spindle cells were uniform in size and arranged in fascicular swirls, and the epithelioid cells formed an adenoid structure surrounded by a basement membrane. In addition, dilated vessels resembling hemangioepithelioma were observed in some areas (Fig. 2C). Immunoprecipitation analysis results were as follows: CK-pan (+) (Fig. 2D), vimentin (+), myogenin (−), CD99 (+) (Fig. 2E), CK20 (−), PAX-8 (−), WT-1 (−), GPC-3 (−), SF1 (−), TLE1 (+), CK7 (ASO+), SMA (−), CD117 (+), S-100 (−), CD34 (−), desmin9 (−), and Ki-67 (20%+). The pathological diagnosis was considered to be hepatic biphasic synovial sarcoma. To further confirm the diagnosis, we sent the pathology section to a higher level hospital for DNA sequencing. The result revealed the fusion gene SS18-SSX1 (+) and finally confirmed the diagnosis of hepatic biphasic synovial sarcoma. The patient recovered well after surgery without complications such as bleeding or biliary fistula and successfully discharged on the 7th postoperative day. The family refused to receive other medication or radiotherapy due to family circumstances. The patient was followed up by telephone and was stable with no recurrence at the date of submission.

## Discussion

Synovial sarcomas originating in the liver are extremely rare, with only 7 cases [5–11] reported in the national and international literature, and a review is summarized in Supplementary Table 1, Additional file. The patient described here is the youngest in all reported cases thus far.

Synovial sarcoma of the liver has an insidious onset, lacks specific clinical symptoms, and has no reported risk factors associated with its development. Patients generally present with abdominal pain with no obvious cause, and most of the lesions have reached a large size at the time of presentation [5–11]. In this case, the patient had abdominal pain, but the main clinical manifestations were nausea and vomiting, which may be due to compression of the stomach by the tumor.

Synovial sarcomas of the liver tend to appear as large aggressive heterogeneous masses on CT. These masses may have hemorrhage and cystic changes, often with calcification, and tend to show uneven moderate or significant enhancement on enhancement CT [12]. MRI is recommended as the test of choice for synovial sarcoma because its higher resolution of soft tissue allows for better evaluation of the lesion. The presence of a “fluid-fluid plane”, a “multicystic mass” or a “triple sign” of both high, equal and low signal on T2WI is indicative of the disease [13]. However, none of these signs are specific, so it is difficult to diagnose hepatic synovial sarcoma by imaging. In our case, the preoperative imaging diagnosis was considered to be hepatic echinococcosis.

Thus, pathology is the only criterion for the diagnosis of hepatic synovial sarcoma. Microscopically, biphasic hepatic synovial sarcoma is composed of different proportions of both spindle cells and epithelioid cells, of which the spindle cells are often uniform in size and tightly arranged in a striated, fasciculated or herringbone shape. Epithelioid cells are often cubic or tall columnar in shape and may be arranged in a glandular, nest-like or papillary structure [1, 10]. Immunohistochemistry is crucial in the diagnosis of hepatic synovial sarcoma. Approximately 90% of synovial sarcomas are positive for keratin, and approximately 60% of cases will be positive for CD99. Additionally, CD34 is negative in almost all synovial sarcomas [1, 10]. Other common pathological manifestations include vimentin (+), S-100 (−), DES (−), SMA (−) and TLE1 (+) [5], all of which are consistent with the immunohistochemical findings of the patient in this case. Because synovial sarcoma has a characteristic chromosomal translocation t(X;18) (p11.2; q11.2) in molecular pathology and produces the fusion gene SYT-SSX with corresponding expression of the fusion protein SS18:SSX [1, 14], it can be detected by FISH and RT–PCR. This has increasingly become an important basis for the diagnosis of synovial sarcoma. Synovial sarcoma of the liver needs to be distinguished from other rare tumors of the liver, such as hepatic sarcoma, malignant mesothelioma, hepatoblastoma, embryonal sarcoma and neuroendocrine tumors.

At present, the main treatment for synovial sarcoma of the liver is a combination of early surgery combined with postoperative chemotherapy, radiotherapy or biological therapy. Because synovial sarcoma of primary origin in the liver is relatively rare, the prognosis for this disease is not yet clear. However, it is promising that as research into the molecular mechanisms of synovial sarcoma continues, biologic therapy may open up new avenues of treatment for this disease.

## Supplementary Information

**Additional file 1.**

## Data Availability

The relevant data and materials pertaining to this study are available upon request.

## Abbreviations

SS: Synovial sarcoma
FISH: Fluorescence in situ hybridization
RT–PCR: Reverse transcription polymerase chain reaction
