# Supplementary material for: Biphasic synovial sarcomas of the liver: a case report and literature review
Source: Diagn Pathol. 2022 Jun 2;17:49. doi: 10.1186/s13000-022-01233-4 (PMC9161578; doi:10.1186/s13000-022-01233-4)
Supplement: Supplementary file 1 — Additional file 1. [file 13000_2022_1233_MOESM1_ESM.docx]

**Table 1. Literature review of 7 cases of synovial sarcomas of the liver**

| Age (y)/Sex | Clinical symptoms | Maximum diameter of tumor (cm) | pathological type | Immunohistochemical features | fusion gene SS18-SSX1 | Treatment | prognosis | Ref |
| --- | --- | --- | --- | --- | --- | --- | --- | --- |
| 42/M | Flatulence, abdominal pain. et al | 11.8 | Unidirectional | VIM(+), CD99(+), Bcl-2(+), PCK (+), CD34(-), EMA(-), S-100(-), CD117(-), DOG1(-), DES(-), Melan-A(-), HMB45(-), SMA(-), GFAP(-). | NR | Surgery | Relapse 3 years later | ^1^ |
| 49/M | Acute abdominal pain | 12 | Biphasic | vimentin(+), CD99(+), Bcl-2(+), EMA(+), CK8(+), CD34(-), EMA(-), S-100(-), SMA(-). | NR | Surgery | NR | ^2^ |
| 21/F | Abdominal pain, emesis, diarrhea. et al | 19 | Unidirectional | CK19(+), CDX2(+), SALL4(+), CK7(+), CD10(+), DPC(+), CD34(-), SMA(-), S-100(-), CD117(-). | (+) | Surgery | No relapse during 3 months follow-up | ^3^ |
| 13/M | Abdominal discomfort | 8.6 | Unidirectional | vimentin(+), BCL-2(+), TLE-1(+), CK7(+), Dog1(-), CD117(-), CD34(-), S-100(-), CD99(-), HMB-45(-). | (+) | Surgery | Relapse 11 months later | ^4^ |
| 44/M | Acute abdominal pain | 14 | Unidirectional | vimentin(+),CD99(+), Bcl-2(+), EMA(+), CKWS(+), CD31(-), CD34(-), S-100(-), HMB45(-), NSE(-). | (+) | Surgery and chemotherapy | Relapse 6 months later | ^5^ |
| 18/F | Abdominal fullness, nausea, emesis.et al | 21 | Unidirectional | AE1(+), AE3(+), CD99(-), CD34(-), CD117(-). | (+) | Surgery | NR | ^6^ |
| 60/F | Acute abdominal pain | 10 | Unidirectional | vimentin(+), BCL-2(+), AE1(-),AE3(-), CAM5.2(-), CK7(-), CK20(-), S-100(-), HMB-45(-), MART-1(-), CD34(-), CD99(-). | (+) | Surgery and chemotherapy | Died 3 months later due to extensive metastasis | ^7^ |
| M: male, F: female, NR: not reported. | | | | | | | | |

[1] Kang z., Min X., Feng Z. et al. Monophasic synovial sarcoma of the liver: a case report[J]. Chinese Journal of Cancer, 2016, 38(12): 949-950. doi:10.3760/cma.j.issn.0253-3766.2016.12.013.

[2] Zhang T., Chen L., Liu Y. et al. Synovial sarcoma of the liver: a case report and literature review[J]. Modern Practical Medicine, 2015, 27(10): 1364-1366+1398. doi:10.3969/j.issn.1671-0800.2015.10.062.

[3] Zajak J., Hladík P., Lischke R. Synovial sarcoma of the liver - case report[J]. Rozhledy v chirurgii : mesicnik Ceskoslovenske chirurgicke spolecnosti, 2015, 94(2): 78-81. doi:

[4] Xiong B., Chen M., Ye F. et al. Primary Monophasic Synovial Sarcoma of the Liver in a 13-Year-Old Boy[J]. Pediatric and Developmental Pathology, 2013, 16(5): 353-356. doi:10.2350/13-04-1327-CR.1.

[5] Janevska V., Filipovski V., Banev S. et al. Synovial Sarcoma of the Liver - A Case Report[J]. Macedonian Journal of Medical Sciences, 2011, 4(2): doi:10.3889/MJMS.1857-5773.2011.0168.

[6] Holla P., Hafez G.R., Slukvin I. et al. Synovial sarcoma, a primary liver tumor – A case report[J]. Pathology - Research and Practice, 2006, 202(5): 385-387. doi:<https://doi.org/10.1016/j.prp.2005.12.006>.

[7] Srivastava A., Nielsen P.G., Dal Cin P. et al. Monophasic synovial sarcoma of the liver[J]. Archives of pathology & laboratory medicine, 2005, 129(8): 1047-1049. doi:10.5858/2005-129-1047-mssotl.
